# Hepatitis B, Hepatitis C, tuberculosis and sexually-transmitted infections among HIV positive patients in Kazakhstan

**DOI:** 10.1038/s41598-021-92688-w

**Published:** 2021-06-29

**Authors:** Ainur Mukhatayeva, Aidana Mustafa, Natalya Dzissyuk, Alpamys Issanov, Bauyrzhan Bayserkin, Sten H. Vermund, Syed Ali

**Affiliations:** 1 Department of Biomedical Sciences, Nazarbayev School of Medicine, Nazarbayev University, Astana, Kazakhstan; 2 Kazakh Scientific Center of Dermatology and Infectious Diseases, Almaty, Kazakhstan; 3 Yale School of Public Health, New Haven, CT USA

**Keywords:** Microbiology, Diseases, Risk factors

## Abstract

In contrast with global trends, HIV prevalence in Kazakhstan and other Central Asian countries has been rising in recent years. In this study, we analyzed hepatitis B (HBV), hepatitis C (HCV), tuberculosis (TB) and sexually-transmitted (STI) co-infections among 500 HIV positive study participants recruited from all regions of Kazakhstan. Among our study participants, 27%, 8%, 2%, and 5% were coinfected with, respectively, HCV, TB, HBV, and STI. A considerable proportion of the study participants was also found with triple or quadruple infections of HCV/TB (12%), TB/STI (0.8%), HCV/STI (2%), HCV/HBV (1%), HBV/TB (0.4%), HBV/STI (0.2%), HBV/HCV/TB (0.4%), HBV/HCV/STI (0.2%), or HCV/TB/STI (0.2%). Strong associations were found of certain age groups, duration of HIV infection, and practices of injection drug use and sexual contact with PLWH, with co-infections of HIV/HCV and HIV/TB. The odds of having death was 4.07 times higher with TB/HIV as compared to other co-infections. Co-occurrence of HIV with HCV, HBV, and TB infections among participants of this study highlights the necessity of regular screening for HCV infection among HIV infected patients, together with implementation of vigilant vaccination protocols against HBV and TB. Additionally, persons who inject drugs especially need to be focused for harm reduction efforts that include opiate substitution therapy, needle or syringe exchange programs, regular screening, and increased availability of ART and direct acting antivirals.

## Introduction

Owing to improved preventive measures, the rates of new HIV infections declined from 2.8 million in 2000 to 1.7 million in 2019 worldwide^1^. Unfortunately, Eastern Europe and Central Asia are exceptions to these trends where, between 2010 and 2018, a 30% increase in new HIV infections has been recorded^2^. The HIV epidemic in Eastern Europe and Central Asia is prevalent mainly in high-risk populations: People who inject drugs (PWID), men who have sex with men (MSM), transgender people, sex workers, prisoners, and their sexual partners comprise 95% of new infections in this region^1,3^. Several factors have contributed to this high incidence of HIV infection^4^. The dissolution of Soviet Union gave rise to a regional financial crisis in 1991^5^, leading to a decline in per capita income, increased rate of unemployment, and consequent renascence of shadow economy. Aftermath of this situation was observed as, among other things, penetration of corruption through the infrastructure responsible for the governance of law, education and healthcare, illegal production and trafficking of opiates leading to noticeable increases in the number of PWID, contributing to the expansion of HIV epidemics in the region^6^. In the beginning, new HIV infections were mostly among PWID, however, the infection quickly bridged into heterosexual populations where it is currently most prevalent^7^.

In Kazakhstan, the number of people living with HIV was 22,000 in 2016, increasing to 24,000 in 2017 and 26, 000 in 2018^8^. In 2018, the prevalence was highest among PWID (7.9%), followed by MSM (6.2%), prisoners (3.5%), and sex workers (1.9%). Trends in the mode of HIV transmission in the country are consistent with those in other Central Asian countries, where the frequency of heterosexual transmission has surpassed that through injection drug use^7^. Overlapping modes of transmission frequently lead to co-infections of human immunodeficiency, hepatitis B, and hepatitis C viruses (respectively, HIV, HBV, and HCV)^9^. In 2018, among people living with HIV (PLWH) in Kazakhstan, 62% and 63% were found coinfected with, respectively, HBV and HCV^10^. Due to the overlapping sexual mode of transmission, sexually transmitted infections (STI) are also known to be common among PLWH^11^.

In this study we report prevalence of coinfection with HBV, HCV, TB, and STI (including gonorrhea, syphilis and trichomoniasis) among a cohort of 500 PLWH in Kazakhstan. We also show an analysis of associations between these co-infections and factors such as gender, risk behaviors, and travel.

## Materials and methods

Ethical approval for this study was obtained from Institutional Research Ethics Committee, Nazarbayev University, Kazakhstan. All methods were performed in accordance with the relevant guidelines and regulations.

We recruited 500 PLWH from all 15 regions of Kazakhstan. The participants were registered with Republican Center for Prevention and Control of AIDS, Almaty, Kazakhstan, asked to signed an informed consent if they were interested in the study and responded to an orally administered questionnaire about their medical history, risk behavior, and existing co-infections, including hepatitis B/C, tuberculosis, and sexually transmitted infections. For diagnosis of HIV and other infections, blood samples were processed by Kazakh Scientific Center of Dermatology and Infectious Diseases, Almaty, where analyses for CD4 + T-lymphocyte count, viral load, and HCV and HBV antibodies were performed using, respectively, BD FACS Count Reagent Kit™ (BD Biosciences, San Jose, USA), AmpliSens® HIV-Monitor-FRT kit (InterLabService, Moscow, Russia), Anti-HCV-ELISA Best kit (Vector-Best, Novosibirsk, Russia), and HBsAg-ELISA Best Kit (Vector-Best, Novosibirsk, Russia). Patients reporting TB symptoms, were tested for TB by chest X-ray and their sputum samples were tested for the presence of Mycobacterium tuberculosis using Xpert® MTB/XDR kit (Cepheid, US). For STIs, study participants were tested at regional or other clinics. In most cases, the following tests were used: For gonorrhea, AmpliSens® Neisseria gonorrhoeae-Test-“FL (InterLabService, Moscow, Russia); for trichomoniasis, AmpliSens® Trichomonas vaginalis-Test-FL (InterLabService, Moscow, Russia), and for syphilis, RecombBest® Treponema pallidum-Summary antibodies Test (Vecor Best, Moscow, Russia).

Statistical analyses were performed using *STATA* version 15.0 statistical software^12^. Categorical variables were summarized in frequencies and percentages. Chi-square, Fisher’s exact, and two-tailed t-tests were used to assess the relationship between each independent categorical and continuous variable, and HCV, HBV, TB, STI/HIV co-infection status. A p-value < 0.05 was used to define statistical significance. Simple and multiple logistic regression analysis was performed to investigate the direction and strength of independent variables on different types of co-infection. Crude and adjusted odds ratios (OR) with 95% confidence interval (CI) were calculated from bivariate and multivariate analysis, respectively.

## Results

### Demographics and risk behavior

**Figure 1:**
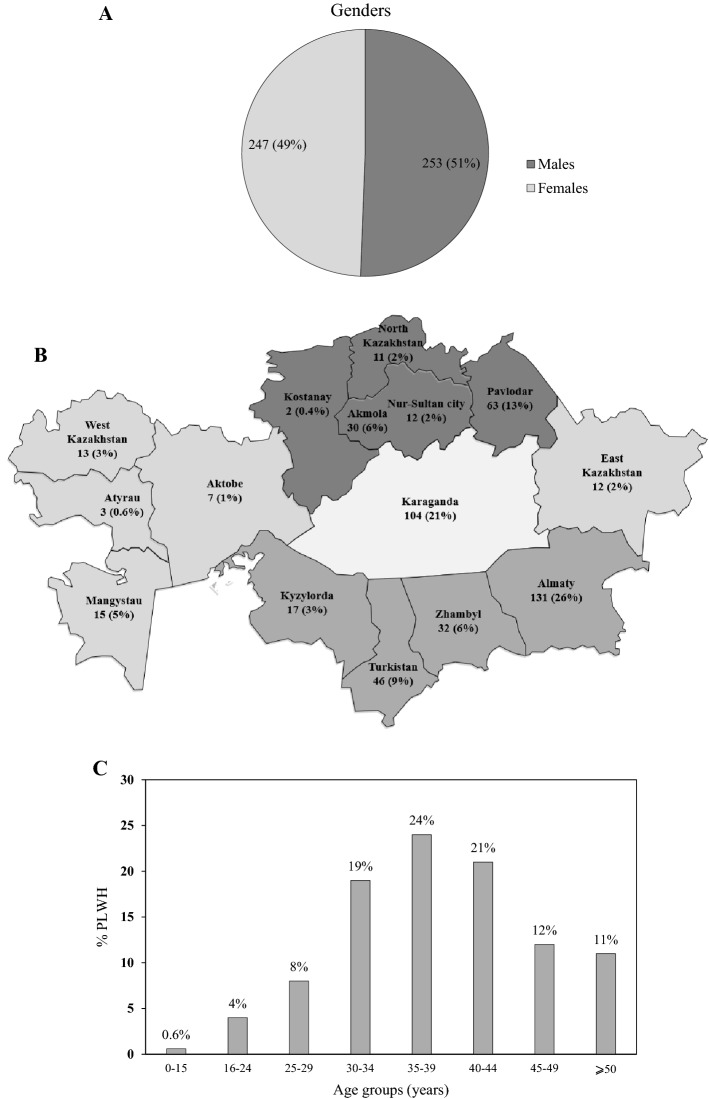

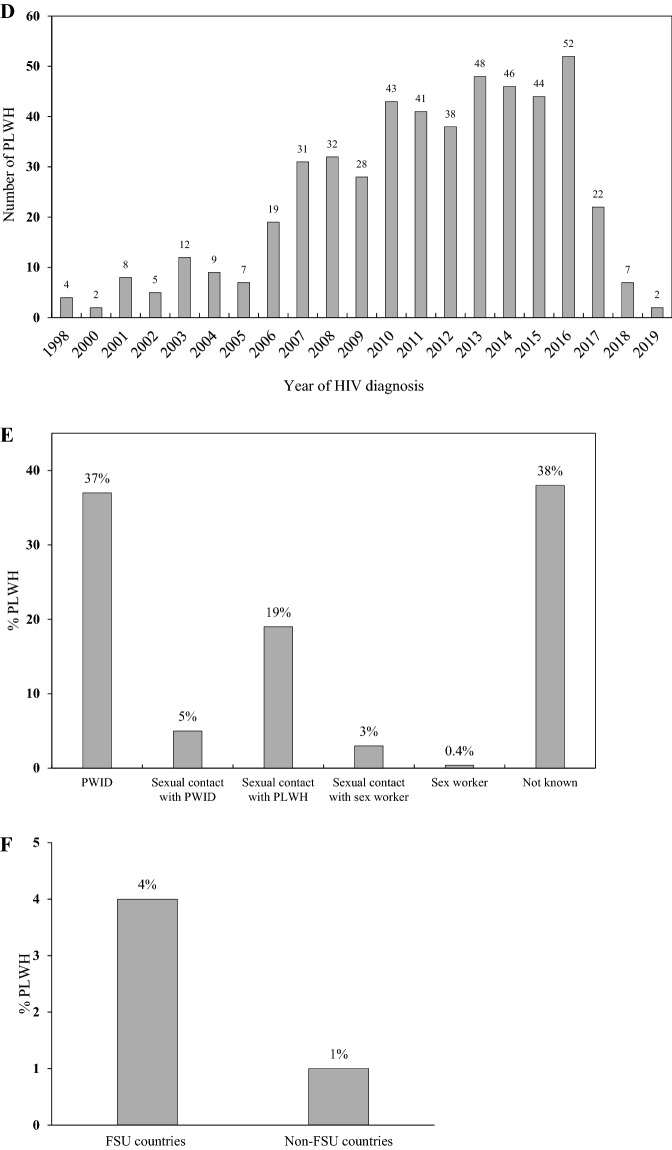
Demographic information of study group with respect to: (**A**) Gender, (**B**) Region of residence, (**C**) Age, (**D**) Year of HIV diagnosis, (**E**) Risk factors, (**F**) Travel history. (**A**) Distribution of males and females among study participants. (**B**) Distribution of HIV positive cases among regions of Kazakhstan, shown as *n* (percentage). (**C**) Representation of various age groups among the study participants. (**D**) Year of HIV diagnosis among the study participants. (**E**) Reported high-risk groups among the study participants. (**F**) Travel history of study participants.

The study participants comprised almost equally of males (51%) and females (49%) (Fig. 1A). Samples were representative of 13 regions of Kazakhstan: Akmola (6%), Aktobe (1%), Atyrau (0.6%), East Kazakhstan (2%), Karaganda (21%), Kostanay (0.4%), Kyzylorda (3%), Mangystau (5%), North Kazakhstan (2%), Pavlodar (13%), Turkistan (9%), West Kazakhstan (3%), Zhambyl (6%); and 2 cities with status of State, i.e., Almaty (26%) and Nur-Sultan (2%) (Fig. 1B and Table 1). The study group comprised patients with mean age of 39 years, while 24% of the patients were aged 35–39 yr, followed by 40–44 yr (21%) and 30–34 yr (19%) (Fig. 1C and Table 1).

**Table 1:** Factors associated with HCV, HBV, TB and STI co-infections among 500 HIV-positive patients.

Variable	Total	HCV	HBV	TB	STI
Total	500	215 (43.0%)	21 (4.2%)	109 (21.8%)	42 (8.4%)
**Sex**					
Female	247 (49.4%)	48 (19.4%)	8 (3.24%)	42 (17.0%)	26 (10.5%)
Male	253 (50.6%)	167^a^ (66.0%)	13 (5.1%)	67^a^ (26.5%)	16 (6.3%)
**Age of HIV diagnosis:**	32.5 ± 9.9	*p* < 0.01^b^	*p* = 0.61	*p* = 0.16	*p* = 0.85
0–24	106 (21.2%)	35 (33.0%)	3 (2.8%)	20 (18.9%)	9 (8.5%)
25–29	112 (22.4%)	47 (42.0%)	9 (8.0%)	19 (17.0%)	10 (8.9%)
30–39	170 (34.0%)	78 (45.9%)	5 (2.9%)	46 (27.1%)	14 (8.2%)
40–49	81 (16.2%)	40 (49.4%)	3 (3.7%)	17 (21.0%)	5 (6.2%)
≥ 50	31 (6.2%)	15 (48.4%)	1 (3.2%)	7 (22.6%)	4 (12.9%)
**Risk factors:**					
PWID	188 (37.6%)	174^a^ (92.6%)	10 (5.3%)	66^a^ (35.1%)	7^a^ (3.7%)
Sexual contact with PWID	23 (4.6%)	6 (26.1%)	0 (0.0%)	2 (8.7%)	4 (17.4%)
Sexual contact with PLWH	95 (19.0%)	26^a^ (27.4%)	2 (2.1%)	17 (17.9%)	10 (10.5%)
Sexual contact with sex worker	13 (2.6%)	7 (53.8%)	1 (7.7%)	4 (30.8%)	2 (15.4%)
Sex worker	2 (0.4%)	0 (0.0%)	0 (0.0%)	0 (0.0%)	0 (0.0%)
Not registered	191 (38.2%)	15^a^ (7.9%)	8 (4.2%)	26^a^ (13.6%)	21 (11.0%)
**Region**					
Akmola	30 (6%)	13 (43.3%)	0 (0.0%)	11 (36.7%)	1 (3.3%)
Aktobe	7 (1.4%)	1 (14.3%)	1 (14.3%)	1 (14.3%)	1 (14.2%)
Almaty	131 (26%)	60 (45.8%)	4 (3.1%)	28 (21.4%)	4 (3.0%)
Atyrau	3 (0.6%)	0 (0.0%)	1 (33.3%)	0 (0.0%)	1 (33.3%)
East Kazakhstan	12 (2.4%)	9 (75.0%)	0 (0.0%)	3 (25.0%)	2 (16.7%)
Karaganda	104 (21%)	37 (35.6%)	3 (2.9%)	20 (19.2%)	1 (1.0%)
Kostanay	2 (0.4%)	1 (50.0%)	1 (50.0%)	0 (0.0%)	0 (0.0%)
Kyzylorda	17 (3.4%)	5 (29.4%)	2 (11.8%)	3 (17.6%)	1 (5.9%)
Mangistau	15 (5%)	5 (33.3%)	0 (0.0%)	3 (20.0%)	3 (20.0%)
North Kazakhstan	11 (2.2%)	7 (63.6%)	0 (0.0%)	2 (18.2%)	0 (0.0%)
Nur-Sultan city	12 (2.4%)	3 (25.0%)	1 (8.3%)	0 (0.0%)	3 (25.0%)
Pavlodar	63 (13%)	31 (49.2%)	3 (4.8%)	17 (27.0%)	7 (11.1%)
Turkistan	46 (9.2%)	21 (45.7%)	3 (6.5%)	11 (23.9%)	5 (10.9%)
West Kazakhstan	13 (2.6%)	7 (53.8%)	1 (7.7%)	5 (38.5%)	1 (7.7%)
Zhambyl	32 (6.4%)	15 (46.9%)	1 (3.1%)	5 (15.6%)	3 (9.4%)
**Duration of HIV infection (years):**	6.4 ± 4.2	*p* < 0.001^b^	*p* = 0.63	*p* < 0.001^b^	*p* = 0.87
0–4	191 (38.2%)	66 (34.0%)	6 (3.1%)	27 (14.1%)	17 (8.9%)
5–9	195 (39.0%)	84 (43.1%)	11 (5.6%)	49 (25.1%)	18 (9.2%)
10–14	89 (17.8%)	46 (51.7%)	3 (3.4%)	21 (23.6%)	6 (6.7%)
15–20	25 (5.0%)	20 (80.0%)	1 (4.0%)	12 (48.0%)	1 (4.0%)
**STI history**					
Yes	42 (8.4%)	12^a^ (28.6%)	2 (4.8%)	5 (11.9%)	-
**Travel history**					
Yes	25 (5.0%)	9 (36.0%)	2 (8.0%)	6 (24.0%)	3 (12.0%)
**Region of travel**					
FSU	19 (76.0%)	7 (36.8%)	2 (10.5%)	6 (31.6%)	2 (10.5%)
Non-FSU	6 (24.0%)	2 (33.3%)	0 (0.0%)	0 (0.0%)	1 (16.7%)
**Life status**					
Died	34 (6.8%)	20 (58.8%)	1 (2.9%)	17^a^ (50.0%)	2 (5.9%)

^a^*p*-value < 0.05 from Chi-square and Fisher’s exact tests.

^b^*p*-value < 0.05 from two-tailed t-test.

HIV, human immunodeficiency virus; HCV, hepatitis C virus; HBV, hepatitis B virus; TB, tuberculosis; STI, sexually transmitted infection; PWID, people who inject drugs; PLWH, people living with HIV; FSU, former Soviet Union.

The majority of study participants were diagnosed with HIV during the year 2010 to 2017 (Fig. 1D). The most common high-risk behavior was injection drug use (37%), followed by sexual contact with PLWH (19%), with PWID (5%), or with sex worker (3%) (Fig. 1E). Only 5% of study participants had traveled abroad, with 76% travelling to former Soviet Union (FSU) countries, including Russia (56%), Kyrgyzstan (12%), Azerbaijan (4%) and Uzbekistan (4%). The remaining 6 patients travelled to non-FSU countries: 3 each to Turkey and China. (Fig. 1F and Table 1).

### Co-infections among PLWH

**Figure 2:**
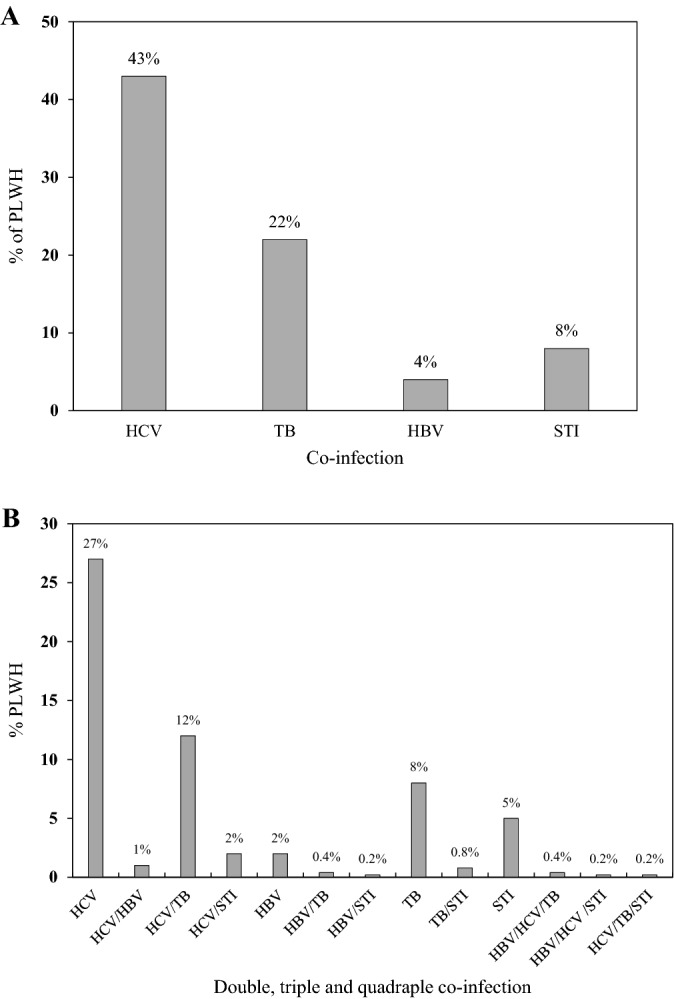
Co-infection: (**A**) Overall co-infection rates and (**B**) double, triple and quadruple infections. (**A**) Occurrence of HCV, TB, HBV, and STI among PLWH study participants. (**B**) Double, triple, and quadruple infections among the study participants.

Overall, out of 500 HIV positive study participants, 215 (43%), 109 (22%), 21 (4%) were found co-infected with respectively, HCV, TB, and HBV (Fig. 2A). Additionally, 42 (8%) were diagnosed positive for sexually-transmitted infections (STI), including gonorrhea, syphilis and trichomoniasis (Table 1 and Fig. 2A). About two-thirds (66%) of total HIV and HCV co-infected patients were males, whereas 19% were females. HIV patients participating in our study carried other co-infections as follows: HIV/HCV (27%), HIV/TB (8%), HIV/HBV (2%), HIV/STI (5%). Furthermore, some participants were triply or quadruply infected with HCV/TB (12%), TB/STI (0.8%), or HCV/STI (2%). Finally, 5 (1%) patients were co-infected with HCV/HBV; 2 (0.4%) with HBV/TB, and 2 (0.4%) with HBV, HCV and TB (Fig. 2B).

Association of various parameters with HCV/HIV co-infection was analyzed using Chi-square test and two-tailed t-test. We found a statistically significant association between HCV/HIV co-infection and the time of presentation, duration of HIV infection, and risk factors, being PWID and sexual contact with PLWH, and presence of STI history (p-value < 0.05) (Table 1). Further, bivariate analysis revealed male sex to be a significant factor for co-infection (OR 8.05; CI 5.35–12.11) (Supplementary Table 1). Additionally, the participants who had HIV diagnosis at the age of 30–39 and 40–49 were, respectively, 1.72 and 1.98 times more likely to have HCV co-infection compared to other patients (OR 1.72; CI 1.04–2.85 and OR 1.98; CI 1.09–3.59) (Supplementary Table 1). Moreover, duration of 10–14 and 15–20 years of HIV infection was revealed to be an independent variable for HCV co-infection (OR 1.99; CI 1.20–3.34 and OR 7.64; CI 2.74–21.27). Among the study participants, the risk of HBV/HIV co-infection was 31 times higher among participants from Kostanay compared to the other regions (OR 31; CI 1.02–941) (Supplementary Table 1).

The significant factors associated with TB/HIV co-infection were found to be gender, age at the time of presentation, duration of HIV infection, or being PWID (p-value < 0.05) (Table 1). Furthermore, the bivariate analysis showed males to have 1.76 times higher rate of TB/HIV co-infection compared to females (OR 1.76; CI 1.14–2.71) (Supplementary Table 1). Moreover, among patients, who had 5–9 and 15–20 years of HIV infection duration, the odds of having TB co-infection was 1.97 and 5.41 higher, respectively, compared to the other groups (OR 1.97; CI 1.17–3.29 and OR 5.41; CI 2.24–13.05) (Supplementary Table 1). On the other hand, in the multivariate model, the male sex was found to be a non-significant independent variable (*p* = 0.70), while being 30–39 years old at the time of HIV diagnosis (OR_adj_ 2.01; CI 1.02–3.93) was a risk factor for TB co-infection in addition to the 5–9 and 15–20 years of HIV infection (OR_adj_ 1.98; CI 1.15–3.40 and OR_adj_ 5.44; CI 1.99–14.82).

Regarding STI/HIV co-infection, chi square test revealed that being PWID and the heterosexual route were significantly associated with STI co-infection (Table 1). Interestingly, bivariate analysis showed that the risk of STI co-infection was decreased by 65% (OR 0.35; CI 0.16–0.74) among participants, who acquired the HIV infection though injection drug use, compared to other modes of transmission (Supplementary Table 1). On the other hand, the multivariate analysis revealed no significant factor having an impact on STI/HIV co-infection.

**Figure 3:**
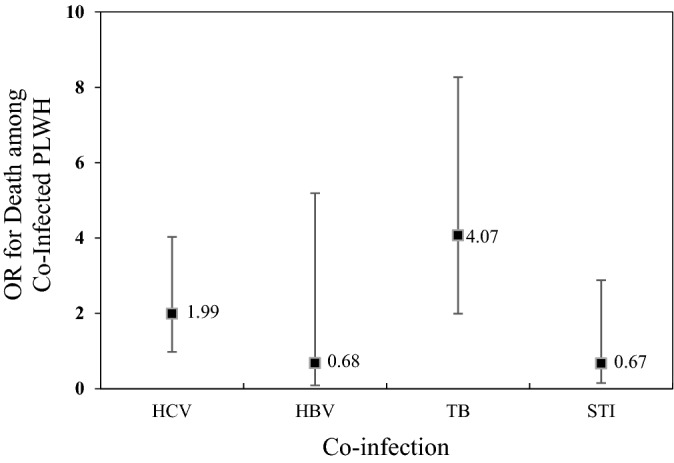
Association between HIV co-infections and death among the study participants; OR, 95% CI.

Finally, assessing the effect of various types of co-infections on death status, the odds of having death among TB/HIV co-infected patients was 4.07 times higher compared to the rest of co-infections (OR 4.07; CI 1.99–8.27) (Fig. 3).

## Discussion

In this study we analyzed 500 blood samples of HIV positive patients from all regions of Kazakhstan, for co-infections and their association with factors such as age, gender, risk behavior, duration of HIV infection, and travel.

### Gender and age

Gender distribution among our study participants was 51% and 49% between, respectively males and female. Since 2003, the number of new HIV cases among women has been gradually increasing in Kazakhstan^13^. Compared to 60% PLWH reported to be male in 2010^14^ and 2019^15^, our study found a shift in this proportion toward females. With respect to age, majority of patients in our study fell in 35-39y age-group (24%), followed by 40-44y (21%), and 30-34y (19%) (Fig. 1C) (Table 1), with cumulatively 64% of the patients falling between the ages 30-44y. According to the progress reports of Republican Center for Prevention and Control of AIDS in Kazakhstan, nowadays there is a transition dynamic in the age distribution of newly HIV infected patients. In 2001, 15% of the all new HIV cases belonged to 15-19y age group^16^. In 2005, the most severely affected age group was 20–29 years old (54%)^13^. In 2015, only 1.5% of newly infected HIV patients belong to 15–19 age group, and the incidence of HIV among 20-29y age group decreased by half (27%). From that period of time, the number of new cases increased in the age group 30–59 y^16^. In 2019, there were 24,389 registered HIV cases with age distribution of HIV prevalence reported as 38%, 30-39y; 34%, 20-29y; and 16%, 40-49y^15^.

### Geographical distribution

Our study group represented samples from 13 regions and 2 cities of Kazakhstan. The largest proportion (26%) of the HIV positive patients in our study was from Almaty region (Fig. 1B), which is also the most populated region of Kazakhstan, representing 21% of the country’s population, comprising people of diverse origins and backgrounds. In other cases, however, the proportion of HIV positive patients did not align with the region/city population. Karaganda and Pavlodar, with, respectively, the second (21%) and third (13%) largest proportion of HIV patients are relatively smaller regions, constituting, respectively, 8% and 4% of the total population in Kazakhstan. This shows an overall high prevalence of HIV in these regions despite smaller numbers of overall inhabitants, owning possibly to particular high-risk practices.

### High risk behavior

The most common mode of transmission among our study participants was through heterosexual sex (57%), followed by injection drug use (41%), and vertical transmission. This is in agreement with the recent trends: a 2019 study conducted with 12,953 participants found heterosexual transmission to be the most common (53%), followed by transmission through injection drug use (40%) and homosexual practice (3%)^15^. According to official statistics, as of 2011, the mode of HIV transmission has shifted from injection drug use to heterosexual sex^17^. Compared to 2006, when 20% infections were found associated with heterosexual contact, this percentage tripled to 67% in 2017^18^. The alarming aspect of this scenario is clearly the bridging of transmission from high-risk groups, such as PWID, to the general heterosexual population.

Similar trends have been recorded in other former Soviet Union countries as well. In Russia, a 2019 study reported more than half (63%) of the HIV patients to have contracted the infection through heterosexual contact, compared to 34% acquiring the infection through injection drug use^19^. A downward trend has been noted in the injection drug use associated transmission: almost 70% in 2004, 60% in 2010, and 39% in 2018^20^. A similar trend has been reported in Uzbekistan^21,22^, where the transmission patterns have evolved in the past two decades among PWID and heterosexual populations with, respectively, 64% and 18% in 2005, 46% and 24% in 2008, and 41% and 42% in 2013^21^. In Ukraine, as well, while an almost equal distribution of transmission was reported among PWID and heterosexual populations in 2007, in 2018, this trend shifted to 30% and 48%, respectively^23,24^.

### Co-infection with HCV, HBV, TB, and STI

We found that in our study group 215 (43%) patients were HIV and HCV co-infected (Fig. 2A). Among these PLWH, 136 (63%) were carrying double co-infection HIV/HCV; 60 (28%) patients were infected with HIV/HCV/TB triple co-infection; 10 (5%) with HIV/HCV/STI; and 5 (2%) with HIV/HCV/HBV (Fig. 2B). Moreover, this study showed that HCV and HIV co-infection was predominant mostly among males aged 30-40y (Table 1). The odds of being co-infected with HCV increased by 2 and 7.6 times for those infected with HIV for, respectively, 10-14y and 15-20y age groups. Our study revealed a significant association between co-infection with HCV and PWID population (Table 1). Globally, an estimated 2.3 million HIV/HCV co-infections are reported, with 1.4 million (59%) of them being PWID. Our finding is in agreement with recent studies showing that the prevalence of HCV co-infection among HIV-infected individuals was highest among PWID in Eastern Europe and Central Asia^25^. This can be due to the overlapping mode of transmission, namely, through needles, for HIV and HCV. In Kazakhstan, HIV/HCV co-infection has been on the rise among PWID^26^. In 2004, a study reported HCV co-infection among 97% HIV-positive patients, whereas that proportion was found to be 63% and 66% in 2005 and 2007, respectively^27^. In a relatively recent study in 2012, HCV co-infection among HIV positive patients was reported to be 53%—higher than, or similar to, the numbers reported from USA (30%), Thailand (50%), Eastern Europe (33%), and Russia (60%)^27^. Such high proportion of blood-borne virus infections in PWID clearly highlights a need for harm reduction efforts in this populations.

In our study group 4.2% of PLWH were co-infected with HBV (Fig. 2A). Among these patients, 10 (48%) were diagnosed with double co-infection (HIV/HBV). Also, some participants were found to have triple or quadruple co-infections: 5 (24%) with HIV/HBV/HCV, 2 (9.5%) with HIV/HBV/TB, and 2 (9.5%) with HIV/HBV/HCV/TB, 1 (4.8%) with HIV/HBV/STI, and 1 (4.8%) with HIV/HBV/STI/HCV (Fig. 2B). Since both HIV and HBV are transmitted by similar routes, i.e., sexually and through injection needles, HIV/HBV infection is common. Globally, 5–20% HIV-positive individuals are reported to be co-infected with HBV^28^, and our findings (4.2%) fall within that range. Since 2019 there were 19,300 registered cases of HBV reported in Kazakhstan^29^. However, due to the wide vaccination coverage number of HBV cases have been decreasing in the country. Over the past 25 years, the incidence of HBV among children and adults has decreased by, respectively,1234.5 and 32 times^30^. In our study, the most affected HIV/HBV age-group was 30-34y (7.4%), whereas no PLWH younger than 24y were diagnosed with HBV (Table 1). It has been reported that HBV co-infection in HIV infected people leads to a greater risk of liver disease progression^31^, increased mortality from AIDS-related events^28^, and poor HIV replication control with combination antiretroviral treatment^32^. Recent studies revealed the importance of employing sensitive biomarkers to identify HBV replicative activity in HIV-infected individuals who test anti-HBc-positive/HBsAg-negative^33^.

Among our study participants, 22% were found co-infected with HIV/TB (Fig. 2A). The odds of TB/HIV co-infection among the study participants were highest among males, who had the HIV infection for 5–9 or 15–20 years. In addition, a significant association was found between PWID and TB/HIV co-infection (Table 1). Furthermore, our analysis showed that odds of death among TB/HIV co-infected individuals were 4 times higher relative to other types of co-infections (Fig. 3). In fact, co-infection with TB among HIV-infected population has been found to be the most common cause of HIV-related deaths^34^*.* According to WHO reports, TB is the most frequent life-threatening opportunistic infection among HIV/AIDS patients. TB co-infection is estimated to affect one-third of the world's HIV-positive population^35^. In HIV-infected people across the world, the prevalence of TB is around 30%^36^. In agreement with these figures, 22% of our HIV positive study participants were infected with TB.

Since 2005, there has been a gradual decrease in TB-associated morbidity and mortality levels in Kazakhstan, but still the country is among the ones, in the WHO European Region, with the highest TB incidence^37^. The probability that people living with HIV will be infected with an active form of tuberculosis is 20–30 times higher than that for HIV negative people^38^. In 2018 the population of Kazakhstan reached 18 million people with 13,361 registered cases of TB, and 730 (5%) coinfected with TB/HIV^39^. Impairment of immunity in HIV infected individuals exposes them to TB infection, with a 20-fold higher risk of reactivation of latent TB^40,]^^41^. Further, susceptibility to TB infection might be increased among PWID due to limited access to health care, compromised housing conditions, overcrowding in places of injection, and poor adherence to treatment^42^.

Among our participants, 8% were co-infected with HIV/STI (Fig. 2A). The most common STIs among participants were syphilis (3.2%), trichomoniasis (3%) and gonorrhea (1.4%). In this study being PWID was significantly associated with STI co-infection (Table 1). This might be due to the fact that PWID may frequently engage in risky sexual behaviors including sexual contact without protection or with multiple partners. According to the statistics of Kazakh Scientific Center of Dermatology and Infectious Diseases, incidence of STI has decreased by 34% between 2017 and 2018, including that of syphilis, gonorrhea, chlamydial infection and urogenital trichomoniasis^43^. MSM and PLWH are at the highest risk for syphilis, according to current surveillance data^44^. According to a comprehensive analysis of research completed across the world, syphilis is found among 9.5% of HIV-positive people^45^. Since both HIV and syphilis are sexually transmitted infections, individuals are frequently found co-infected with both. These viruses can be transferred through oral, vaginal, or anal mucosa^46^ ^47^. While only 3.2% were co-infected with syphilis, compared to other STIs, the prevalence of syphilis among our study participants was the highest. In comparison, 3% of the participants were found co-infected with trichomoniasis. The global prevalence of these co-infections varies depending on the country^48^. Some studies highlight the significance of trichomoniasis in HIV positive people due to the potential role of trichomoniasis in increasing the risk of HIV transmission^48–50^. Among our study participants, co-infection of gonorrhea (1.4%) was to a lesser degree compared to what is globally reported. In the US, for instance, median point prevalence of HIV/gonorrhea co-infection is reported to be 9.5%^51^.

### Socio-economics factors influencing access to healthcare

Overall there are 17 AIDS centers throughout the country, with one center per region. Kazakhstan, unlike most other former Soviet Union nations, allocates the budget for HIV treatment without the help of international contributors^52^. The Republican AIDS Center, Almaty, conducts quarterly monitoring and prevention programs carried out in the country, among key populations (PWID, RS, MSM), with the help of trust points (TP), friendly offices (FO), and non-governmental organizations (NGOs). The tools for monitoring and evaluation are electronic online systems of authorities and the National Database of Customers of Professional Programs^53^. Needle and syringe programs (NSPs) have played a critical role in containing the HIV pandemic across the world by offering HIV prevention information, syringes, and condoms to high-risk populations^54^. NSPs are widely available for PWID in Kazakhstan as well, with over 137 locations all over the country^18^. Moreover, since Kazakhstan is one of the nations affected most by the tuberculosis pandemic^55^, recently integrated approaches for TB and HIV care have been implemented, including that all patients infected with TB must be tested for HIV and vice versa^56^. Additionally, pregnant women are tested for HIV twice amid pregnancy, and there are no laws prohibiting PLWH from entering the country, while they are legally protected from discrimination^52^. Finally, PLWH are entitled to get ARV treatment free of charge. The Joint United Nations Program has devised a 90–90-90 program with the goal of lowering new HIV infections to 500,000 per year by 2020^57,58^. In 2019, among 33,000 PLWH, 18,000 were receiving treatment, while 14,000 PLWH had achieved viral load control, and Kazakhstan's progress towards the 90–90-90 target had moved up to 77–68–80^59^.

## Conclusion

We have found a high prevalence of HBV, HCV, and TB co-infections among the HIV-positive patients in our study, It is known that coinfection with HIV can promote the progression of the HBV- and HCV-associated liver disease, accelerate the development of fibrosis, and increase the risk of hepatic carcinoma^60,61^. The significant association between HIV/HCV co-infection among participants in this study highlights the necessity of regular screening for HCV infection among HIV infected patients. Clearly there is an urgent need to prevent HIV and HCV transmission among PWID by harm reduction, including opiate substitution therapy, needle or syringe exchange programs, regular HCV screening, increased availability of ART and direct acting antivirals against HCV^62,63^.The prevalence of TB-HIV coinfection is highest in African and Asian countries^64^. Furthermore, the incidence of TB-HIV coinfection is increasing in Kazakhstan^65^. Implementation of vigilant vaccination protocols against TB and HBV is one effective way to prevent these coinfections among PLWH.

## Supplementary Information

Supplementary Table 1.
