# Author Correction: Hepatitis B, Hepatitis C, tuberculosis and sexually-transmitted infections among HIV positive patients in Kazakhstan

**DOI:** 10.1038/s41598-021-97673-x

**Published:** 2021-09-07

**Authors:** Ainur Mukhatayeva, Aidana Mustafa, Natalya Dzissyuk, Alpamys Issanov, Bauyrzhan Bayserkin, Sten H. Vermund, Syed Ali

**Affiliations:** 1 Department of Biomedical Sciences, Nazarbayev School of Medicine, Nazarbayev University, Astana, Kazakhstan; 2 Kazakh Scientific Center of Dermatology and Infectious Diseases, Almaty, Kazakhstan; 3 Yale School of Public Health, New Haven, CT USA

Correction to: *Scientific Reports* 10.1038/s41598-021-92688-w, published online 29 June 2021

The original version of this Article contained an error, where diagnostic tests for STI were omitted.

As a result, in the Materials and Methods section, now reads:

"For STIs, study participants were tested at regional or other clinics. In most cases, the following tests were used: For gonorrhea, AmpliSens® Neisseria gonorrhoeae-Test-“FL (InterLabService, Moscow, Russia); for trichomoniasis, AmpliSens® Trichomonas vaginalis-Test-FL (InterLabService, Moscow, Russia), and for syphilis, RecombBest® Treponema pallidum-Summary antibodies Test (Vecor Best, Moscow, Russia)."

Additionally, p value is erroneously mentioned as p = 0.xx in the Results section under ‘Co-infections among PLWH’ subsection,

“On the other hand, in the multivariate model, the male sex was found to be a non-significant independent variable (p=0.xx), while being 30–39 years old at the time of HIV diagnosis (OR_adj_ 2.01; CI 1.02–3.93) was a risk factor for TB co-infection in addition to the 5–9 and 15–20 years of HIV infection (OR_adj_ 1.98; CI 1.15–3.40 and OR_adj_ 5.44; CI 1.99–14.82).”

now reads:

“On the other hand, in the multivariate model, the male sex was found to be a non-significant independent variable (p=0.70), while being 30–39 years old at the time of HIV diagnosis (OR_adj_ 2.01; CI 1.02–3.93) was a risk factor for TB co-infection in addition to the 5–9 and 15–20 years of HIV infection (OR_adj_ 1.98; CI 1.15–3.40 and OR_adj_ 5.44; CI 1.99–14.82).”

The original Article has been corrected.

