# Supplementary material for: Hepatitis B, Hepatitis C, tuberculosis and sexually-transmitted infections among HIV positive patients in Kazakhstan
Source: Sci Rep. 2021 Jun 29;11:13542. doi: 10.1038/s41598-021-92688-w (PMC8241865; doi:10.1038/s41598-021-92688-w)
Supplement: Supplementary file 1 — Supplementary Table 1. [file 41598_2021_92688_MOESM1_ESM.docx]

**TITLE**

Co-Infections of HCV, HBV, TB and STI among HIV Positive Patients in Kazakhstan

**AUTHORS**

Ainur Sanaubarova^1^, Aidana Mustafa^1^, Natalya Dzissyuk^2^, Alpamys Issanov^1^, Bauyrzhan Bayserkin^2^, Sten Vermund^3^, Syed Ali^1^

**AFFILATIONS**

^1^Department of Biomedical Sciences, Nazarbayev School of Medicine, Nazarbayev University, Astana, Kazakhstan

^2^Kazakh Scientific Center of Dermatology and Infectious Diseases, Almaty, Kazakhstan

^3^Yale School of Public Health, New Haven, CT, USA

**Supplementary Table 1.** Logistic analysis on HCV, HBV, TB and STI co-infection factors among 500 HIV-positive patients

| **Variable** | **OR (95% CI) of HCV** | **OR (95% CI) of HBV** | **OR (95% CI) of TB** | **OR (95% CI) of STI** |
| --- | --- | --- | --- | --- |
| Gender:   - Female - Male | Reference  8.05^a^ (5.35-12.11) | Reference  1.62 (0.66-3.98) | Reference  1.76^a^ (1.14-2.71) | Reference  0.57 (0.30-1.10) |
| Age of infection:   - 0-24 - 25-29 - 30-39 - 40-49 - ≥50 | Reference  1.47 (0.84-2.55)  1.72^a^ (1.04-2.85)  1.98^a^ (1.09-3.59)  1.90 (0.84-4.29) | Reference  3 (0.79-11.39)  1.04 (0.24-4.44)  1.32 (0.26-6.72)  1.14 (0.11-11.41) | Reference  0.88 (0.44-1.76)  1.60 (0.88-2.89)  1.14 (0.55-2.35)  1.25 (0.47-3.32) | Reference  1.05 (0.41-2.71)  0.97 (0.40-2.32)  0.71 (0.23-2.20)  1.60 (0.46-5.59) |
| Region by oblasts:   - Akmola - Aktobe - Almaty city - Atyrau city - East Kazakhstan - Karaganda - Kostanay - Kyzylorda - Mangystau - North Kazakhstan - Nur-Sultan city - Pavlodar - South Kazakhstan - West Kazakhstan - Zhambyl | Reference  0.22 (0.02-2.04)  1.11 (0.50-2.46)  -  3.92 (0.88-17.50)  0.72 (0.32-1.65)  1.31 (0.75-22.93)  0.55 (0.15-1.94)  0.65 (0.18-2.38)  2.62 (0.64-10.61)  0.44 (0.10-1.94)  1.27 (0.53-3.04)  1.01 (0.40-2.54)  1.53 (0.41-5.64)  1.15 (0.42-3.14) | Reference  5.17 (0.28-94.50)  0.98 (0.11-9.05)  15.5 (0.69-350.63)  -  0.92 (0.09-9.17)  31^a^ (1.02-941.00)  4.13 (0.35-49.28)  -  -  2.82 (0.16-49.01)  1.55 (0.16-15.53)  2.16 (0.22-21.79)  2.58 (0.15-44.70)  - | Reference  0.29 (0.03-2.71)  0.47 (0.20-1.10)  -  0.58 (0.13-2.59)  0.41^a^ (0.17-0.99)  -  0.37 (0.09-1.58)  0.43 (0.09-1.87)  0.35 (0.06-1.87)  -  0.64 (0.25-1.61)  0.54 (0.20-1.48)  1.08 (0.28-4.13)  0.32 (0.09-1.07) | Reference  -  0.91 (0.10-8.48)  14.5 (0.64-328.46)  5.8 (0.47-71.07)  3.43 (0.42-27.71)  -  1.81 (0.11-30.97)  7.25 (0.68-76.86)  -  9.67 (0.89-104.82)  3.63 (0.43-30.89)  3.54 (0.39-31.89)  2.42 (0.14-41.87)  3 (0.29-30.56) |
| Duration of HIV infection (years):   - 0-4 - 5-9 - 10-14 - 15-20 | Reference  1.44 (0.96-2.18)  1.99^a^ (1.20-3.34)  7.64^a^ (2.74-21.27) | Reference  1.85 (0.67-5.12)  1.09 (0.27-4.48)  1.29 (0.15-11.19) | Reference  1.97^a^ (1.17-3.29)  1.72 (0.91-3.27)  5.41^a^ (2.24-13.05) | Reference  1.05 (0.52-2.10)  0.75 (0.29-1.98)  0.43 (0.05-3.37) |
| STI history:   - Yes | 0.50 (0.25-1.01) | 1.16 (0.26-5.14) | 0.46 (0.18-1.20) | - |
| Travel History:   - Yes | 0.73 (0.32-1.70) | 2.09 (0.46-9.50) | 1.14 (0.44-2.93) | 1.52 (0.44-5.32) |

*^a^ significant associations from bivariate logistic analysis*

*HIV, human immunodeficiency virus; HCV, hepatitis C virus; HBV, hepatitis B virus; TB, tuberculosis; STI, sexually transmitted infection; OR, odds ratio; CI, confidence interval.*
